# Antibacterial efficacy of silver diamine fluoride as a root canal irrigant

**DOI:** 10.1002/cre2.222

**Published:** 2019-07-16

**Authors:** Ebtissam M. Al‐Madi, Manar A. Al‐Jamie, Noura M. Al‐Owaid, Amal A. Almohaimede, Albandary M. Al‐Owid

**Affiliations:** ^1^ Department of Restorative Dentistry, College of Dentistry King Saud University Riyadh Saudi Arabia; ^2^ Department of Dentistry Ministry of Health Riyadh Saudi Arabia; ^3^ College of Dentistry Princess Nourah bint AbdulRahman University Riyadh Saudi Arabia

**Keywords:** antimicrobial, chlorhexidine, endodontics, *Enterococcus faecalis*, irrigation, nanoparticles

## Abstract

**Objectives:**

Conventional disinfectants and medicaments have not been able to achieve and maintain sterilization of root canals. The purpose of the study was to evaluate the antibacterial efficacy of 3.8% silver diamine fluoride (SDF) in comparison with 2% chlorhexidine (CHX) against *Enterococcus faecalis* biofilm.

**Materials and methods:**

Extracted human teeth were used to make 70 dentin discs that were then inoculated with *E. faecalis* to generate a 3‐week‐old biofilm model. The discs were subjected to treatment with 3.8% SDF, 2% CHX, sodium hypochlorite (NaOCl), or saline for 10 min. After exposure, the dentin discs were examined with a confocal laser scanning microscope to verify the percentage of live versus dead cells within the biofilm. Univariate one‐way analysis of variance and Tukey‐honestly significant difference post hoc analysis (*p* < .05) were performed to detect significant differences.

**Results and conclusion:**

The NaOCl group showed the greatest percentage of dead cells (62.26%) among all groups (*p* < .05). The SDF group showed a significantly higher percentage of dead cells (57.39%) than the 2% CHX and saline groups (*p* < .05). SDF possessed higher antimicrobial activity than 2% CHX against *E. faecalis* biofilms.

## 1 INTRODUCTION

Effective root canal treatment depends on appropriate instrumentation, irrigation, and obturation. Silver diamine fluoride (SDF) is an anticariogenic material with a high fluoride release capacity. It has been proposed to be a very effective agent, especially in pediatric dentistry. It has also been used in endodontic medicaments. Endodontic irrigation plays an important role in root canal therapy and is considered a fundamental step in instrumentation. The irrigant should eradicate the remaining pulp tissue, bacteria, and debris that might persist after meticulous biomechanical preparation (Du et al., 2014; González‐Luna IV et al., 2016).

Endodontic infection is a polymicrobial infection, and obligate anerobic bacteria are the main cause of pulpal and periapical inflammation in primary infections. In persistent infections, different microorganisms are associated with intraradicular and extraradicular infection (Narayanan & Vaishnavi, 2010). *Enterococcus faecalis* is a Gram‐positive facultative anaerobic microorganism that presents in cases of chronic recurrent periapical infection and failed endodontic therapy (Evans, Davies, Sundqvist, & Figdor, 2002; González‐Luna IV et al., 2016). *Enterococcus faecalis* can attack dentinal tubules and survive in them for a long period of time (Love, 2001), attach to dentin and develop a biofilm under various environmental conditions (George, Kishen, & Song, 2005), resist root canal disinfectants, and survive difficult conditions within endodontically treated teeth (Rôças, Siqueira, & Santos, 2004). There have been limited attempts to eradicate *E. faecalis* biofilm with commonly used root canal irrigants in the past. However, conventional disinfectants and medicaments have not been able to achieve and maintain sterilization of root canals (Kishen & Haapasalo, 2010).

SDF is an anticariogenic material with a high fluoride release capacity. It has been proposed to be a very effective agent, especially in pediatric dentistry. It has also been used in endodontic medicaments (Mathew, Madhusudhana, Sivakumar, Venugopal, & Reddy, 2012).

The null hypothesis tested is that there was no difference in the antibacterial efficacy of 3.8% SDF Ag (NH_3_)2F (SDF) in comparison with 2% chlorhexidine (CHX) and 5.25% sodium hypochlorite (NaOCl) when these agents were used as endodontic irrigants to reduce the percentage of live *Enterococcus faecalis* bacteria in biofilms of infected root canals. The goal of this study was to examine the antibacterial effectiveness of SDF in comparison with CHX against *E. faecalis* biofilm by using confocal laser scanning microscopy (CLSM).

## 2 MATERIALS AND METHODS

### 2.1 Preparation of dentin specimens

Human teeth with single roots were collected after applying the following inclusion criteria: roots with no prior endodontic treatment, fracture lines, anatomical irregularities, or curvatures. The teeth were decoronated, and a diamond disc was used to section off the apex of the roots. A low‐speed saw (IsoMet 2000 Precision Saw; IsoMet, Buehler, IL) rotating at 1,000 rpm in a buccolingual direction under water cooling was used to split the roots in the middle. Dentin discs with dimensions of 6 x 8x 0.5mm (width x length x thickness) were prepared, and 17% ethylenediamineetracetic acid (EDTA) was applied to all discs to remove the smear layer. The discs were then rinsed for 1 min with sterile saline. Finally, all discs were sterilized in an autoclave at 121°C for 20mn and stored in brain heart infusion (BHI) broth (Bukhary & Balto, 2017; Mathew et al., 2012).

### 2.2 Bacterial inoculation

The sterilized dentin discs were arranged in culture plate wells and inoculated with *E. faecalis* suspension under anaerobic conditions at 37ºC for 21 days. Inoculation was repeated with a fresh culture every 72 h. After inoculation, phosphate‐buffered saline (PBS) was used to rinse the specimens to eliminate non‐adherent bacteria as well as the culture medium. The dentin discs were checked arbitrarily with CLSM (Nikon Eclipse Ti‐E, Mississauga, Canada) to confirm the existence of biofilms of *E. faecalis* on the surface of the dentin.

### 2.3 Treatment of infected specimens

A total of 70 dentin discs were split into the experimental groups A and B (each *n* = 20) and control groups C and D (each *n* = 15). The discs in group A were irrigated with 3.8% SDF, while those in group B were irrigated with 2% CHX. The specimens in group C (positive control) were irrigated with 5.25% NaOCl, and those in group D (negative control) were irrigated with sterile saline.

Each sample was submerged in 2 ml of the test solution for 10 min. The dentin discs were then rinsed with 5 mL of PBS and neutralized with neutralizing agents. Sodium hypochlorite was deactivated with 5% sodium thiosulfate solution, while silver diamide was neutralized with normal saline, and chlorohexidine was neutralized by a combination of 0.3% lecithin and 3% Tween (Bukhary & Balto, 2017; Mei et al., 2013). All irrigation procedures were performed by the same operator under aseptic conditions and at room temperature.

### 2.4 Confocal laser scanning microscopy analysis

The dentin discs were washed with 2 mL of sterile PBS and stained for 30 min with the LIVE/DEAD fluorescence dye (Invitrogen Molecular Probes). This dye is composed of two dyes: Calcein‐AM and ethidium homodimer‐1 that are applied to distinguish between dead and live cells. Bacteria with damaged membranes stain red, while those with intact cell membranes stain green. A new mix for each disc was prepared immediately prior to microscopic evaluation. The specimens were washed with 2 mL of PBS to eliminate the surplus dye. The samples were viewed using the CLSM 10x/0.45 dry lens.

Dual‐channel imaging was used concurrently to exhibit the red and green fluorescence representing dead and live cells, respectively. The emission/excitation wavelengths were 494/517 nm for calcein‐AM and 528/617 nm for ethidium homodimer‐1. Images of the biofilms were quantitated and analyzed.

The results were analyzed statistically and summarized using univariate one‐way analysis of variance and Tukey‐honestly significant difference post hoc analysis, for mean comparisons between groups at a significance level of *p* <.05 by using SPSS version 11.5.

## 3 RESULTS

**Figure 1:**
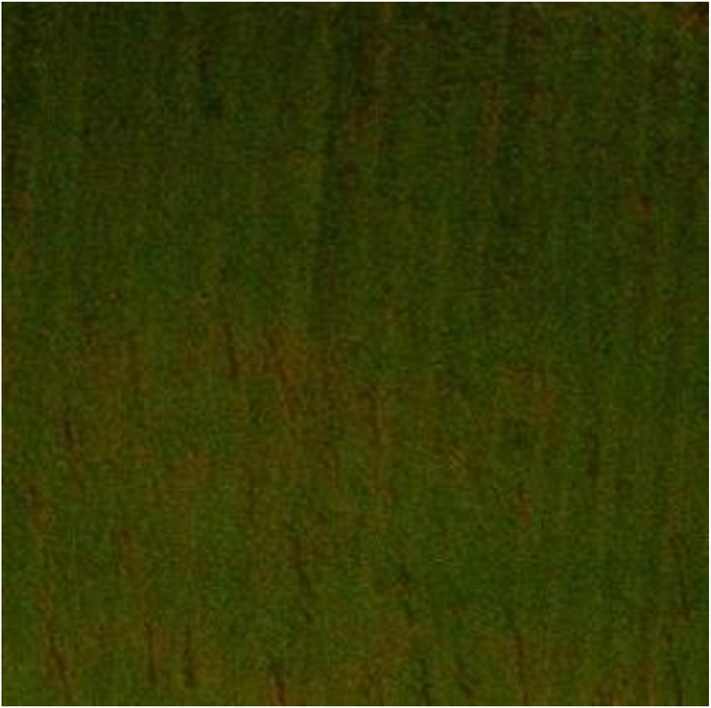
Confocal laser scanning microscopic images of 3‐week‐old E. faecalis‐infected dentin discs

A homogenous dense biofilm was seen on the surface of the dentin discs, which confirmed the presence of *E. faecalis* (Figure 1). A significantly larger percentage of dead cells was apparent in the group exposed to SDF (57.39%), followed by the 2% CHX (53.39%) samples. The positive control group (5.25% NaOCl) showed the largest percentage of dead cells (62.26%) in comparison to the experimental groups (*p* < .05). The smallest percentage was detected in the negative control (48.28%). Statistically significant differences were found between the percentages of dead cells in all groups (*p* < .05) (Table 1).

**Table 1:** Mean and standard deviation values of live and dead cells after exposure to irrigant solutions for 10 min

Groups	Mean dead cells	Mean live cells	Standard deviation
3.8% SDF	57.39*	42.60*	3.63
2% chlorhexidine	53.39*	46.60*	2.80
5.25% sodium hypochlorite	62.26*	37.73*	7.41
Saline	48.28*	51.71*	6.35

Abbreviation: SDF: silver diamine fluoride.

* The mean difference is significant at the 0.05 level.

**Figure 2:**
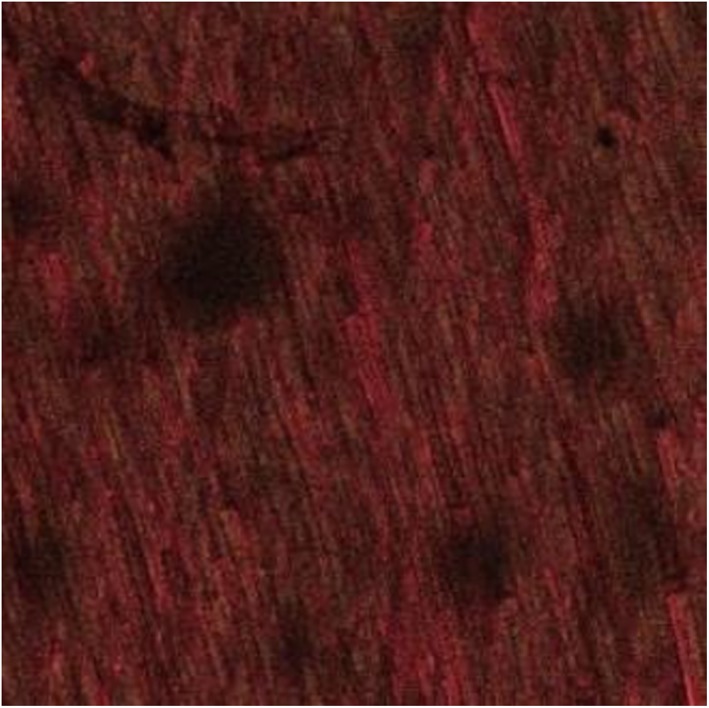
Dentin discs after exposure to 3.8% silver diamine fluoride for 10 min

**Figure 3:**
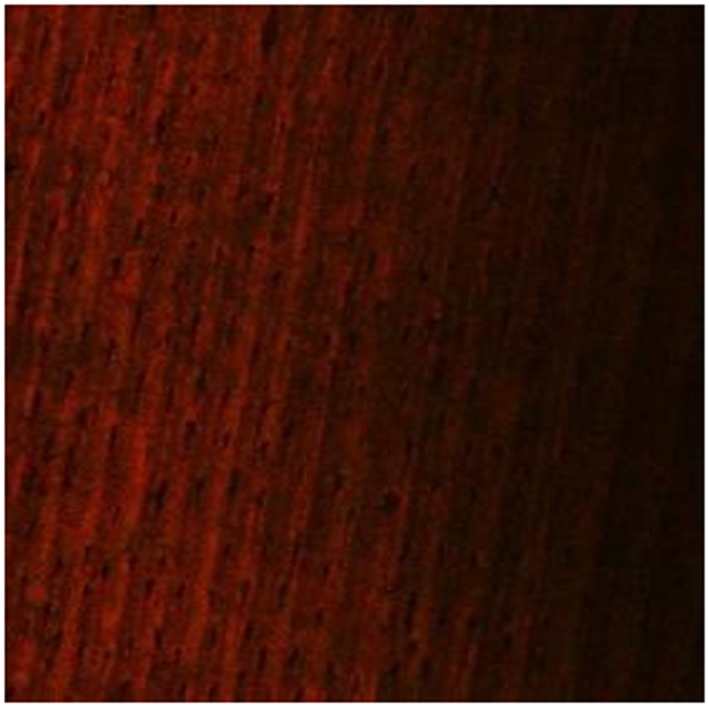
Dentin discs after exposure to 5.25% NaOCl for 10 min

**Figure 4:**
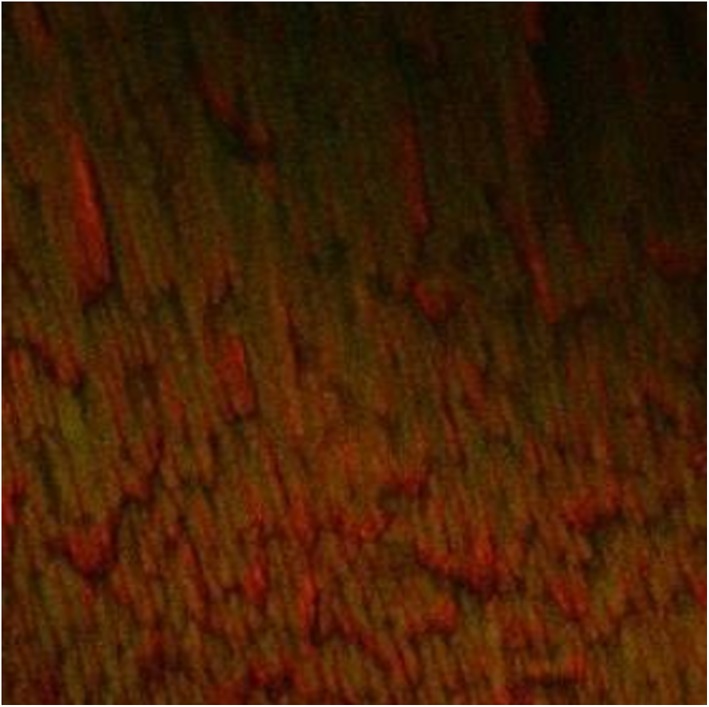
Dentin discs after exposure to 2% chlorhexidine for 10 min

**Figure 5:**
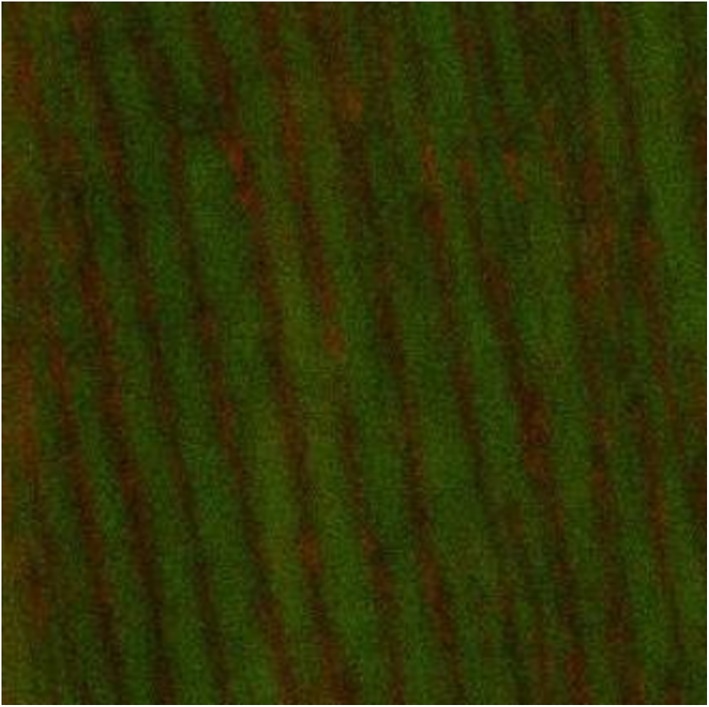
Dentin discs after exposure to saline for 10 min

The images of the 3‐week‐old *E. faecalis* biofilm revealed significant removal of bacterial biofilm with a few residual dead cells near the dentin surfaces in the 3.8% SDF (Figure 2) and 5.25% NaOCl groups (Figure 3). The biofilm exposed to 2% CHX showed variable levels of structural damage and multiple bacterial cells attached to the dentin surfaces (Figure 4). In contrast, the saline group exhibited many live bacteria attached to dentin surfaces and an intact biofilm layer (Figure 5).

## 4 DISCUSSION

The goal of this study was to examine the antibacterial effectiveness of SDF, when compared with CHX against *E. faecalis* biofilm by using confocal laser scanning microscopy.

Endodontic infections are typically biofilm‐mediated; thus, cleaning and irrigation of the root canal is a crucial aspect in the treatment of these infections and for preventing recurrence (González‐Luna et al., 2016). The structure of the biofilm and the characteristics of the inhabitant microorganisms provide the biofilm resistance against external influences such as antimicrobial agents. In addition, prolonged exposure of bacteria to disinfectants might lead to resistance to consequent exposure (Evans et al., 2002). Thus, effective biofilm elimination and obliteration of the inhabitant bacteria are crucial to root canal disinfection.

*Enterococcus faecalis* was used in the present study because it shows a low prevalence in primary endodontic infections (4–40%) and a high prevalence in persistent infections (24–77%) (Baca, Junco, Arias‐Moliz, González‐Rodríguez, & Ferrer‐Luque, 2011). *Enterococcus faecalis* has the capability to survive and resist treatment with the commonly used irrigant solutions. It has a dentinophilic nature that allows it to bind to the dentin surface and permeate up to 400 μh, proliferate, and recolonize in the root canal system (Bukhary & Balto, 2017; Mathew et al., 2012). Dentin discs were used due to their suitability as a substrate for biofilm formation. However, newly generated biofilms cannot represent the complete structural development or extracellular polymeric matrix production in biofilms, since the bacteria are in the exponential growth phase. Therefore, 3‐week‐old *E. faecalis* biofilms appear to be more resistant to disinfecting solutions than young biofilms, and a 3‐week period of incubation was used in the present study to ensure maturation of the biofilm (Bukhary & Balto, 2017). The infected dentin discs in this study were exposed to the irrigant solutions for 10 min, previous studies reported that exposure to the disinfectant solution for 10 min is considered the maximum effective time to kill a 3‐week‐old *E. faecalis* biofilm in a dentin infection model (Bukhary & Balto, 2017).

Antibacterial cationic nanoparticles such as silver nanoparticles (AgNP) show substantial antibacterial activity against biofilms. AgNPs show multiple antibacterial mechanisms such as adherence and penetration into the bacterial cell wall, leading to a loss of integrity of the bacterial cell membrane and cell wall permeability (Rai, Deshmukh, Ingle, & Gade, 2012). It is thought that the positively charged nanoparticles electrostatically interact with negatively charged bacterial cells, resulting in altered cell permeability, intracellular leakage, and, ultimately, bacterial death (Rabea, Badawy, Stevens, Smagghe, & Steurbaut, 2003). The AgNP solution has been recommended as an alternative to root canal irrigating solutions not only for its strong bactericidal potential but also for its biocompatibility, especially at lower concentrations (Gomes‐Filho et al., 2010).

SDF is an effective anticariogenic agent with high fluoride release capacity. It can increase the hardness of the tooth surface and remineralize it (Chu, Lo, & Lin, 2002; Yokoyama, Kimura, Matsumoto, Fujishima, & Miyazaki, 2001). It has been presented as a reasonable caries‐preventive agent that is both efficient and safe, meeting the criteria of l potential but also for its biocompatibility, especially at lower concentrations (Gomes‐Filho et al., 2010).

SDF is an effective anti‐cariogenic agent with high fluoride release capacity. It can increase the hardness of the tooth surface and re‐mineralize it (Chu et al., 2002; Yokoyama et al., 2001). It has been presented as a reasonable caries‐preventive agent that is both efficient and safe, meeting the criteria of “World Health Organization's Millennium goals” (Rosenblatt, Stamford, & Niederman, 2009). A 3.8% SDF solution was prepared for intracanal irrigation. This solution was a 1:10 dilution of the initial 38% SDF solution that had been utilized for root canal disinfection (Hiraishi, Yiu, King, Tagami, & Tay, 2010). However, the antibacterial effect of this solution against endodontic pathogens has not been explored significantly. Hiraishi et al. studied the effect of 3.8% SDF and NaOCl on in vitro *E. faecalis* biofilm. They reported that 3.8% SDF showed 100% efficiency against *E. faecalis* after a direct 60‐min exposure (Hiraishi et al., 2010). In the current study, we compared SDF with CHX irrigation and used a lower exposure time to further verify the effectiveness of SDF. CHX is characterized by a cationic molecular component that adheres to the regions of the cell membrane that have a negative charge and leads to cell lysis. However, it does not have the ability to eradicate debris or organic tissue (Davis, Walton, & Rivera, 2002). Other studies have shown the minimal effectiveness of other agents (NaOCl and CHX) in eliminating bacteria in biofilm after the first 3 min, with only small amounts of new bacteria being killed between 10 and 30 min (Du et al., 2014). Therefore, the SDF exposure time was limited to 10 min in this study.

The present study revealed that the percentage of dead cells in the SDF group was greater than that in the CHX group (57.39%). SDF eradicated most of the *E. faecalis* in the biofilm, which is in agreement with the results of prior studies (Hiraishi et al., 2010; Mathew et al., 2012). However, previous studies noted a greater antibacterial effect of SDF than that shown in the current study. This might be attributable to the differences in antibacterial evaluation procedures, the type of bacteria, and the reduced exposure time.

The current study revealed that the antibiofilm effect of 2% CHX (53.39% dead cells) was lower than that of SDF (57.39%). This might be because the organic matter of the biofilm had inactivated the cationic bisbiguanides as well as the partial penetration of the extracellular matrix of the biofilm (Spijkervet et al., 1990; Yamaguchi et al., 2013). Similar results were reported in earlier studies in which CHX showed limited antibacterial effects (Agrawal et al., 2013; Kangarlou Haghighi, Tashfam, Nasseri, Dianat, & Taheri, 2013). In contrast, one previous study reported that CHX showed greater antibacterial activity than the other experimental irrigants (Mathew et al., 2012).

The percentage of dead cells was significantly greater in the NaOCl group (62.26%) compared with the other groups (*p* < .05), with almost complete eradication and dissolution of the *E. faecalis* biofilm. These results are similar to previous studies that have proven the potency of 5.25% NaOCl in the elimination of biofilms (Dunavant, Regan, Glickman, Solomon, & Honeyman, 2006; Hiraishi et al., 2010; Kim & Shin, 2017; Ruiz‐Linares, Aguado‐Pérez, Baca, Arias‐Moliz, & Ferrer‐Luque, 2017). NaOCl is the most frequently used solution during root canal therapy. It has strong antimicrobial activity and can dissolve organic tissue (Arias‐Moliz, Ferrer‐Luque, Espigares‐García, & Baca, 2009; Zehnder, 2006). The potent antimicrobial effects of NaOCl are due to its ability to destroy organic tissue and damage the extracellular matrix of the biofilm. Chlorine has the ability to affect a wide range of microbes, including fungi and viruses, and oxygen eradicates anaerobic bacteria. However, the use of NaOCl has various inherent disadvantages, such as an unpleasant smell and taste, high toxicity, and extreme corrosiveness to metals. Furthermore, 40% to 60% of the root canals irrigated with NaOCl still retain bacteria in the main canal, and its clinical performance is inferior to its effects in vitro (Kim & Shin, 2017). Since NaOCl does not have residual antimicrobial activity, persistent microorganism regrowth can still occur (Baca et al., 2011). The limited antibiofilm effect of saline (48.28%) was in agreement with previous studies that exhibited a significantly lower level of antibiofilm effects of saline than in the other tested groups (Kaushik, Rehani, Agarwal, Kaushik, & Adlakha, 2013; Narayanan & Vaishnavi, 2010).

SDF has been reported to discolor radicular dentin (Hiraishi et al., 2010). This was not apparent in the current study. This might be due to the low dilution used in the current study. Although this issue is not expected to raise esthetic concerns, it should be evaluated in the future.

## 5 CONCLUSIONS

Within the limits of the current in vitro study, it may be concluded that SDF possessed higher antimicrobial activity than 2% CHX against *E. faecalis* biofilms. These findings have great potential for improving intracanal irrigation, given the lower toxicity of SDF. The biocompatibility of the solution should be further investigated. in vivo studies are needed to confirm the conclusions of the current study.

## FUNDING

This research did not receive any specific grant from funding agencies in the public, commercial, or not‐for‐profit sectors.

## CONFLICTS OF INTEREST

The authors have no financial affiliation (e.g., employment, direct payment, stock holdings, retainers, consultantship, patent licensing arrangements, or honoraria) or involvement with any commercial organization with direct financial interest in the subject or materials discussed in this manuscript nor have any such arrangements existed in the past 3 years.
